# Multiparametric Ultrasound Approach Using a Tree-Based Decision Classifier for Inconclusive Focal Liver Lesions Evaluated by Contrast Enhanced Ultrasound

**DOI:** 10.3390/jpm11121388

**Published:** 2021-12-20

**Authors:** Tudor Voicu Moga, Ciprian David, Alina Popescu, Raluca Lupusoru, Darius Heredea, Ana M. Ghiuchici, Camelia Foncea, Adrian Burdan, Roxana Sirli, Mirela Danilă, Iulia Ratiu, Teofana Bizerea-Moga, Ioan Sporea

**Affiliations:** 1 Advanced Regional Research Center in Gastroenterology and Hepatology, Department of Gastroenterology and Hepatology, “Victor Babeş” University of Medicine and Pharmacy, 300041 Timişoara, Romania; moga.tudor@yahoo.com (T.V.M.); alinamircea.popescu@gmail.com (A.P.); heredeadarius@gmail.com (D.H.); anamaria.ghiuchici@gmail.com (A.M.G.); foncea.camelia@gmail.com (C.F.); burdanghitaadrian@gmail.com (A.B.); roxanasirli@gmail.com (R.S.); mireladanila@gmail.com (M.D.); ratiu_iulia@yahoo.com (I.R.); isporea@umft.ro (I.S.); 2 Electronics and Telecommunications Faculty, “Politehnica” University of Timișoara, 300006 Timișoara, Romania; ciprian.david@upt.ro; 3 Center for Modeling Biological Systems and Data Analysis, Department of Functional Sciences, “Victor Babes” University of Medicine and Pharmacy, 300041 Timisoara, Romania; 4 Department of Pediatrics—1st Pediatric Discipline, “Victor Babeș” University of Medicine and Pharmacy, 300041 Timisoara, Romania; teofanabizerea@yahoo.com

**Keywords:** multiparametric ultrasound, liver fibrosis, contrast-enhanced ultrasound, focal liver lesions

## Abstract

Background: Multiparametric ultrasound (MPUS) is a concept whereby the examiner is encouraged to use the latest features of an ultrasound machine. The aim of this study was to reanalyze inconclusive focal liver lesions (FLLs) that had been analyzed via contrast enhanced ultrasound (CEUS) using the MPUS approach with the help of a tree-based decision classifier. Materials and methods: We retrospectively analyzed FLLs that were inconclusive upon CEUS examination in our department, focusing our attention on samples taken over a period of two years (2017−2018). MPUS reanalysis followed a three-step algorithm, taking into account the liver stiffness measurement (LSM), time–intensity curve analysis (TIC), and parametric imaging (PI). After processing all steps of the algorithm, a binary decision tree classifier (BDTC) was used to achieve a software-assisted decision. Results: Area was the only TIC-CEUS parameter that showed a significant difference between malign and benign lesions with a cutoff of >−19.3 dB for washout phenomena (AUROC = 0.58, Se = 74.0%, Sp = 45.7%). Using the binary decision tree classifier (BDTC) algorithm, we correctly classified 71 out of 91 lesions according to their malignant or benignant status, with an accuracy of 78.0% (sensitivity = 62%, specificity = 45%, and precision = 80%). Conclusions: By reevaluating inconclusive FLLs that had been analyzed via CEUS using MPUS, we managed to determine that 78% of the lesions were malignant and, in 28% of them, we established the lesion type.

## 1. Introduction

Ultrasound (US) is a widely used method for diagnosis. It is easy to use, non-invasive, and does not irradiate the patient, and thus can be repeated as many times as necessary. Because all US machines have grayscale imaging (B-mode) and Doppler, these modes are used as the first line of imaging diagnosis. With the evolution of ultrasound engineering, however, multiple other options have emerged, increasing the utility of US. Thus, new ultrasound devices have the ability to perform multiple elastographic methods, enabling us to assess fibrosis. Moreover, with the introduction of contrast medium (contrast-enhanced ultrasound (CEUS)), the paradigm of ultrasound indication has changed, especially for focal liver lesions (FLLs).

By providing contrast for US, broad spectrums of features have been implemented in US machines, allowing us to quantitatively analyze organ perfusion in terms of time and intensity (time–intensity curve and parametric imaging analysis). However, CEUS examination for FLL might have some drawbacks related to the echoic window, liver cirrhosis, and the examiner’s experience [1,2,3].

The new imaging options offer a broader perspective of the examined structures and have led to the creation of a concept called multiple parametric ultrasound (MPUS) [4], a concept that is also used in the literature for assessing other pathologies. MPUS is used successfully to describe parathyroid lesions, thyroid nodules, prostate cancer, breast lesions, and chronic kidney disease. All of these new features have boosted US to the level of other contrast imaging techniques, which were considered superior to US not so long ago. Of all the organs, the liver has probably been the most privileged and, in the context of focal liver evaluation, the MPUS concept brings considerable advantages. With the amount of information that US can offer, the time needed to perform a complete examination with all the features applied has increased. Considering this fact, the conceptualization of US might be changed in MPUS evaluation [4,5]. There is still little data available in the literature regarding the MPUS concept in FLL evaluation to the best of our knowledge, and none regarding the use of MPUS in inconclusive FLLs that had been evaluated by CEUS. With so much information to process, dedicated software can streamline the job and classifiers can be applied to the MPUS steps in order to facilitate medical decisions.

The aim of our study was to reassess inconclusive focal liver lesions (FLL) that had been evaluated by CEUS with the help of the MPUS concept, using a binary decision tree classifier in order to highlight the malignancy of the lesions as the first endpoint, and later on to identify the lesion type as the second endpoint.

## 2. Materials and Methods

### 2.1. Study Design and Population

**Figure 1:**
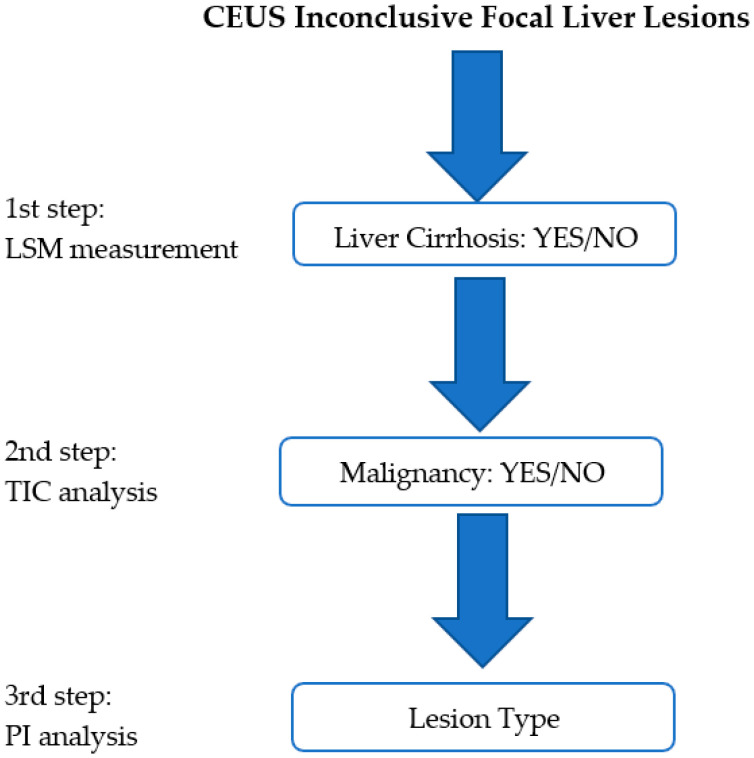
The proposed MPUS steps. The first step consists of liver stiffness evaluation (LSM). In the second step, malignancy is ruled in/out with the help of time–intensity curve (TIC) analysis, while in the third step, the lesion type is analyzed with the help of the parametric imaging (PI) features.

We reevaluated all of the inconclusive focal liver lesions (FLL) that had been evaluated using contrast enhanced ultrasound (CEUS) during a two-year period (2017–2018). All of the lesions included in the study had a second line imaging method—contrast-enhanced computer tomography (CE-CT), with contrast-enhanced magnetic resonance imaging (CE-MRI) or biopsy—that was considered as the reference method. US and CEUS examination were performed by US experts [6] with more than 10 years of experience of using CEUS. All FLLs were evaluated with a single US machine—a LOGIQ E9 from General Electric Healthcare (Chalfont, St. Giles, UK). During the post-processing analysis, the operator was blinded to the final diagnosis. The reevaluation was based on three steps that were carried out with the help of the features offered by the US machine. The steps consisted of liver stiffness measurement (LSM), time–intensity curve analysis (TIC), and parametric imaging (PI), hereinafter referred to as multiparametric ultrasound (MPUS) evaluation (Figure 1).

Inclusion criteria: Patients older than 18 years of age, with focal liver lesions detected via grayscale ultrasound, but without the possibility of a final characterization and for whom contrast-enhanced ultrasound (CEUS) was inconclusive as a final diagnosis, according to the European Federation of Societies for Ultrasound in Medicine and Biology’s (EFSUMB) [1] recommendations.

Exclusion criteria: Patients for whom CEUS was suggestive of a final diagnosis or for whom the CEUS examination was considered a failure, whereby, for example, there was an inappropriate examination and focus (lesion too deep or too shallow), a lack of vascular phases, a bad echoic window, or inappropriate lesion tracking and portal vein thrombosis.

This retrospective study was conducted according to the Declaration of Helsinki and approved by our ethics committee (no. 9/09.05.2016). Written informed consent was obtained from each patient prior to CEUS examination.

### 2.2. Multiparametric Ultrasound Steps

The MPUS steps used the ultrasonographic features imbedded in the newly advanced ultrasound machines. Being a retrospective study, some of the data were collected from the patient file (LSM) and some were achieved through the post-processing features of the CEUS examination (TIC; PI).

**Figure 2:**
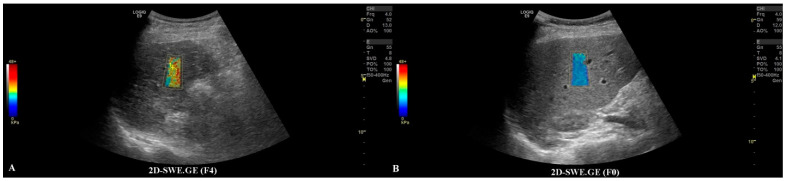
In frame (**A**), we have depicted a cirrhotic liver evaluated by the 2D-SWE.GE technique, where the color-coded map points out a reddish color, compared with frame (**B**), where the color-coded map depicts a blue color, meaning the absence of fibrosis.

The first step was to exclude or confirm liver cirrhosis (F = 4) by measuring liver stiffness (LS). It was possible to complete this step by using the 2D-SWE.GE technique offered on a LOGIQ E9 scanner with a C1-6-D probe [7] or by using vibration-controlled transient elastography (VCTE), using the M and XL probes (FibroScan^®^, Echosens, Paris, France) [8]. The measurements were performed according to the EFSUMB guidelines for liver elastography [9]. All of the patients that were evaluated in our department for liver lesions were, by default, screened for liver fibrosis via non-invasive means; thus, this information could be found in the patients’ files (Figure 2).

**Figure 3:**
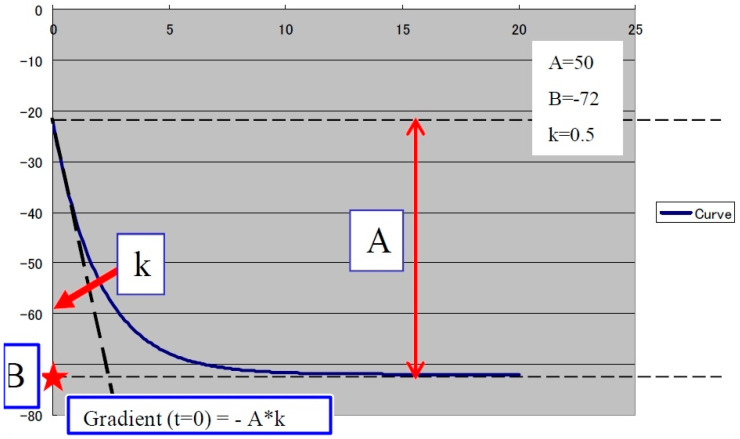
The curve fitting for washout: F(t) = Aexp(−kt) + B, as explained in the graphic above, where exp(−kt): decreasing function for “washout”. For a larger k, the intensity decreases quickly. B: the minimum intensity at t = infinity. The gradient at t = 0 was calculated using “−A*k”. A is the difference between B and the intercept intensity at t = 0. MSE: mean square error between the actual data and the fitting curve.

The second step was to quantitatively analyze the tissue perfusion in the late phase of CEUS evaluation using the TIC feature, and to highlight the presence of FLL washout. CEUS examination was performed with the C1-6 convex probe following the CEUS guidelines for the liver. The contrast agent used was SonoVue^®^ (Bracco SpA, Milan, Italy), given as an intravenous bolus of 1.7 mL of SonoVue, followed by 10 mL of saline [1,2]. The late phase (>120 s) of each lesion was reassessed and evaluated via time–intensity curve analysis. The curve fitting parameters for the washout in the bolus injection were time to peak, area under the curve, maximum gradient, and curve gradient, which were evaluated in order to depict the slightest washout phenomena of the lesion. Curve fitting for washout was calculated with the following formula: Washout: F(t) = Aexp(−kt) + B (as explained in Figure 3) [10]. Two regions of interest were selected, one outlining the lesions and the second reference region near the parenchyma, which is possible to perceive at the same depth. All of the lesions’ washout phases were evaluated using TIC.

**Figure 4:**
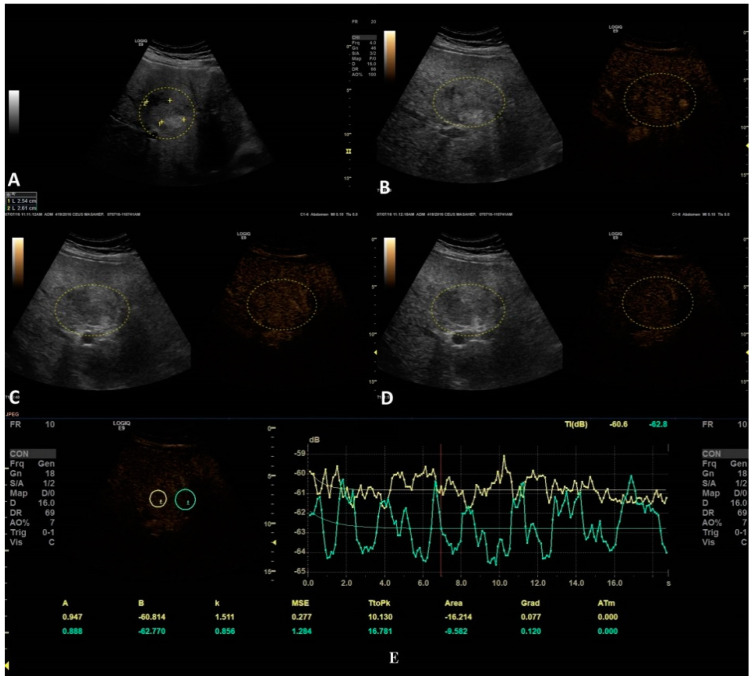
In this setting, a benign lesion was assessed, as depicted in B-mode in frame (**A**). In frames (**B**–**D**) are depicted the vascular arterial, portal, and late phases of CEUS with different enhancement characteristics, respectively. In frame (**D**), the enhancement pattern is not recognizable; thus, TIC analysis (**E**) was applied. The results did not show us any objective washout phenomena (the graphs are not intersecting one another).

**Figure 5:**
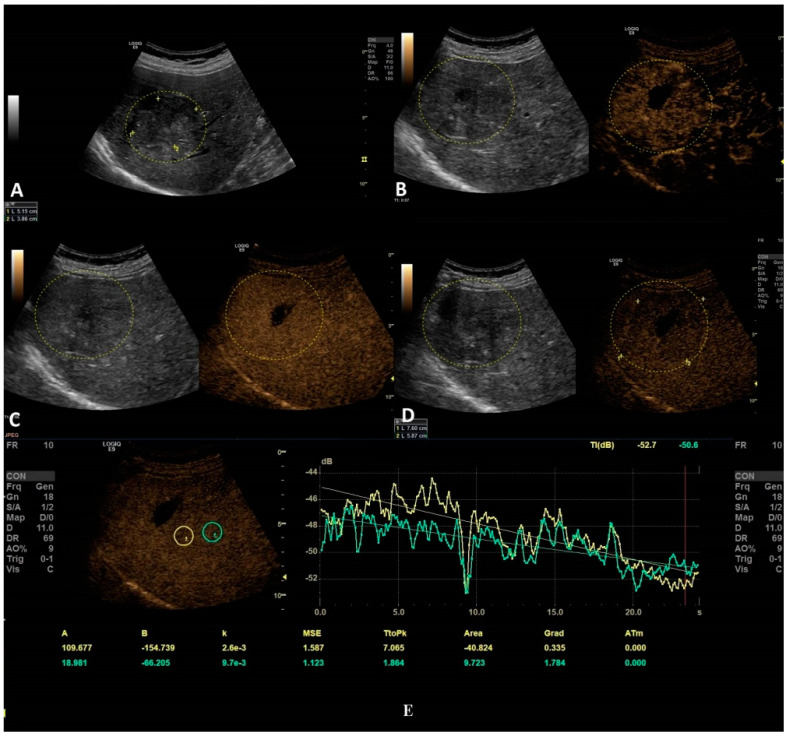
In this setting, a malignant lesion was assessed, as depicted in B-mode (**A**). CEUS evaluation is depicted in frames (**B**–**D**). In frame (**D**), the enhancement pattern is not recognizable; thus, TIC analysis (**E**) was applied. The results revealed objective washout phenomena (the graphs are intersecting one another).

The last step consisted of applying the parametric imaging feature from GE-LOGIQ E9 [11] that enabled, according to a color-coded map, an evaluation of the perfusion kinetics of the lesion. This allowed us to better observe the enhancing pattern and the hyper- and hypo-enhanced areas of an FLL with the help of a colored display. Parametric imaging was used in the third step, with the intention to correctly differentiate the type of lesion after the malignancy was established in step two. The arterial phase (10–20 to 30–45 s) of each inconclusive lesion was subject to PI and classified according to the typical enhancement pattern explained in Table 1 [1] (Figure 4 and Figure 5). 

**Figure 6:**
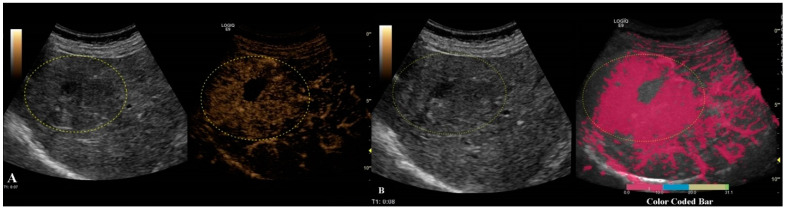
Arterial phase of CEUS is depicted in frame (**A**). In frame (**B**), parametric imaging was applied, thus better documenting the arrival time of bubbles by using color coding, as shown in the color-coded bar.

Different colors were used to define different arrival times, as shown in Figure 6. As a result, a color-coded map was superimposed over the B-mode image.

### 2.3. Statistical Analysis

Data analysis was performed using MedCalc v19.3 from MedCalc Software Ltd., Ostend, Belgium. Categorical variable comparisons were performed using the chi-square or Fisher exact test, and continuous variables were evaluated with Student’s *t*-test or the Mann–Whitney test. A *p*-value < 0.05 was considered to be statistically significant. For the TIC analysis, the factors associated with malignancy were assessed using linear regression analysis. For the evaluation of the performance of the MPUS score, receiver operating characteristic (ROC) analysis was used. Optimal cutoff values were taken from the corresponding receiver operating characteristic curves. Sensitivities, specificities, positive and negative predictive values, and accuracy were calculated.

### 2.4. Binary Decision Tree Classifier

**Figure 7:**
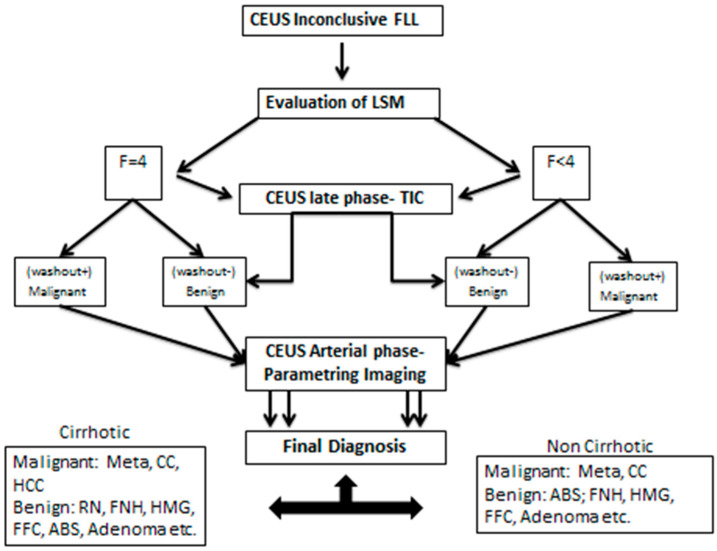
The binary tree decision classifier with the decision steps and possible alternatives.

After processing all of the steps of the algorithm and clustering all the data, a binary decision tree classifier (BDTC) was used to facilitate software-assisted decision making. This process employs a decision tree model to use MPUS data to arrive at conclusions about the presence of cirrhosis, the malignancy of lesions, and the type of lesions. The binary decision tree consists of three steps. First, fibrosis is tested and compared with a reference of F4 and below. The second step is a comparison of the TIC value with a predefined value. In the third step, subjective parametric imaging is used. At this point, the vascular pattern at the CEUS arterial stage is analyzed. Based on these decisions, the binary tree decides on one or another of the following lesions: metastasis, FNH, HCC, etc. (as explained in Figure 7).

## 3. Results

Baseline characteristics are presented in Table 2.

From the 91 inconclusive lesions, the reference methods established that 34 were HCC, 13 were metastasis, seven were haemangioma, seven were regenerative nodules, five were focal fatty alterations, three were fatty free areas, four were cholangiocarcinoma, two were abscesses, five were adenomas, and 11 were benign lesions (complex biliary cysts, parenchymal infarction areas, and vascular abnormalities).

The only TIC-CEUS parameter that showed significant differences between malign and benign lesions was AREA (−25.08 ± 37.98 and −7.08 ± 42.6, *p* = 0.04). The parameter AREA is in fact the area under the curve. The curve fitting algorithm is applied and a curve is obtained according to the intensity observed. Some examples of fitted curves are shown in both Figure 4a,b, where parameter AREA is the area under the fitted curve. Since the washout is characterized by a smaller intensity of activity, the observed curve will fall and hence the fitted curve will fall, too. This fact leads to a smaller AREA parameter. This phenomenon allows us to impose a threshold on the AREA parameter in order to detect the washout. We also tested A (the difference between B and the intercept intensity at t = 0), B (the minimum intensity at t = infinity), k (the gradient at t = 0, calculated by “−A*k”), TtoPk (time to peak intensity from the start frame to the end frame), but without any statistical significance (all *p*-values > 0.05) (Table 3).

Regression analysis showed a significant relationship between the area in lesion and the area in parenchyma (*p* = 0.02) metrics. The regression equation (y = −12.21 + 0.16 x) showed that the coefficient for the area in parenchyma is 0.16. The coefficient indicates that for every additional unit for the area in parenchyma metric, the area in lesion will increase by an average of 0.16 dB.

The best cutoff value for the area in lesions for predicting malignancy and washout in inconclusive lesions was >19.3 dB with an AUROC of 0.58, an Se of 74.0%, and an Sp of 45.7% (Table 4).

**Figure 8:**
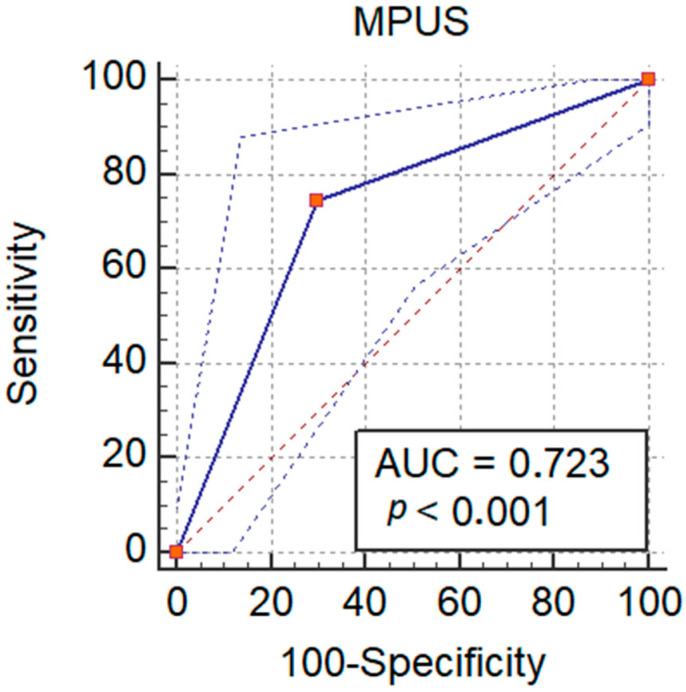
Performance of MPUS in detecting malignant liver lesions.

B-mode ultrasonography performed well for detecting malignant lesions, with an AUROC of 0.52 and a sensitivity of only 15.6%, while elastography had an AUROC of 0.64 and a sensitivity of 74.5%. By adding the value of elastography, along with the cutoff value of the area in lesions from the CEUS-TIC analysis, we increased the performance to an AUROC of 0.72 (Figure 8) and a sensitivity of 90.2% for detecting malignant lesions with washout (Table 3). MPUS correctly classified 66/91 lesions, with an accuracy of 72.3%.

### BDTC Algorithm

The BDTC algorithm is based on what was proven in the statistical analysis. The model composed of the parameter from CEUS-TIC analysis with a cutoff value of <−19.3 dB and elastography, with patients being split into non-cirrhotic (<F4) and cirrhotic patients (F4), combined with the enhanced type and the type of washout, gave us the BDTC algorithm for diagnosing inconclusive liver lesions. With the BDTC algorithm, we correctly classified 71/91 lesions according to their malignant or benignant status, with an accuracy of 78.0%.

When comparing the statistical analyses, there were no differences between the accuracies in diagnosing benign and malignant lesions (72.3% vs. 78.0%, *p* = 0.39), but we correctly diagnosed the type of lesion in 20 inconclusive lesions, with a detection rate of 28%. In the malign versus benign scenario, we found that most of the lesions that will benefit from this diagnosis algorithm are HCC (23 detected as malignant), metastases (nine detected as malignant) and regenerative nodules (five detected as benign). From the 20 focal liver lesions that were correctly diagnosed, nine were HCCs, six were metastases, two were FNHs, and two were fatty free areas.

Concerning the performance of the previously presented BDTC in the cases of benign/malignancy results, we obtained 44 good detections as malignant lesions from a total of 55, and 27 out of 35 good detections for benign lesions. These detections provided us the following performance scores: accuracy = 78%, sensitivity = 62%, specificity = 45%, precision = 80%, and F1 score = 69.8%.

## 4. Discussion

FLLs are often detected in daily practice when performing a routine grayscale abdominal ultrasound. The new features implemented in the newer ultrasound machines allow the examiner to gain a better understanding of the structural and functional changes of the lesions. Therefore, the role of the ultrasound examination is to assess functional changes by analyzing vascularization through both quantitative and qualitative means and structural changes by measuring the stiffness both of the liver and the focal liver lesion. In the current study, a MPUS-based classifier was constructed to help reassess inconclusive FLLs that had been evaluated using CEUS. The MPUS classifier used, as well as CEUS and liver elastography, a CEUS quantification feature (TIC) that enables tissue perfusion analysis and a further tool, PI, which empowers the examiner to better document the contrast in arrival time and pattern using a color-coded map. A binary decision tree classifier was conceived that helped us rule in or rule out malignancy in the first step and the type of lesion in the second step.

The introduction of contrast medium in liver ultrasounds was a game changer for hepatologists worldwide. FLLs can be accurately evaluated with CEUS [12,13,14,15] and now even have some advantages when compared with CE-CT or CE-MRI, such as the possibility to perform multiple evaluations on the same patient (with no side effects or specific contraindications) in a real-time assessment with comparable performance. Being an intravascular agent, microvascularization will be improved, and thus the washout phenomena better depicted [1]. However, there are situations in which CEUS cannot be confidently relied upon when evaluating an FLL. Factors such as liver cirrhosis, the dimension of the lesion, histological differentiation, vascular abnormalities, a lack of an acoustic window, or even focal fatty sparing might lead to misdiagnosis during CEUS [1,3,16,17].

Multiparametric conceptualization was first used in magnetic resonance imaging due to its numerous sequences and functional imaging. The concept was mainly used for prostate cancer evaluation and staging due to the lack of specificity of conventional methods (prostate-specific antigens, digital rectal examination, and transrectal ultrasound biopsy). Prostate MRI provides an excellent assessment of the standard anatomical sequence, and the additional information from the contrast medium offers high accuracy for tumor detection localization and staging, thus influencing related management [18].

The idea of MPUS in US was originally considered by Cantisani [19] for differentiating the malignancy of thyroid nodules using multiparametric ultrasound and quantitative elastosonography. Ninety-seven patients with thyroid nodules were evaluated through both color Doppler ultrasound and elastographic means (Elasto-Q, Toshiba) before surgery. The two methods were then compared in terms of hypoechogenicity, irregular margins, and suspicion of halo features, with researchers concluding that elastosonography is a valuable asset for predicting malignancy. In a later editorial [4,5], the terminology of multiparametric ultrasound examination was preferred to that of classical US examination, with the authors pointing to the new developments in US that have expanded the amount of information an examiner could produce with little extra effort.

To date, there has only been one report about multiparametric ultrasound in focal liver lesions [20]. Grgurevic et al. reviewed the literature regarding MPUS in hepatology and proposed an MPUS-based practical algorithm for practitioners when managing chronic liver disease and FLLs. For diffuse chronic liver disease, Grgurevic et al., with the help of B-mode US, Doppler US, and liver stiffness measurements (based on the transient elastography and 2D-SWE methods), recommended a pathway for when chronic liver disease is suspected. When evaluating a “de novo” FLL, the authors divide tumors into solid and cystic tumors. We did not consider this necessary, keeping in mind the low incidence of malignant cystic lesions in daily practice [1,21] and the ease of diagnosing a simple cyst using standard US. Furthermore, the authors include the Doppler evaluation and the clinical context in the first step of the algorithm. We did not rely on Doppler evaluation, intending to use CEUS evaluation later on; however, “the clinical context” must eventually be included as a key element in all “de novo FLL” algorithms. Undoubtedly, a cancerous or cirrhotic context would certainly influence the examiner when depicting an FLL. In our study, we chose to dichotomize only cirrhotic from non-cirrhotic patients because we could quantify the information using liver stiffness measurements. Patients with cancer history would have already been evaluated with cross-sectional imaging for staging purposes, a situation that is beyond the scope of the MPUS concept. In the same algorithm, the authors evaluating tumor stiffness offer a cutoff for “liver elsatography malignancy prediction (LEMP)” that would strongly suggest malignancy. Despite the potential benefit of tumor stiffness evaluation [22] in some tumors (such as HCC), we believe that further studies are needed in order to establish a cutoff that will take into consideration the dimension, type, and liver background of a tumor; nevertheless, LEMP could be integrated into further algorithms. Aside from the proposed practical algorithm, we investigated more CEUS parameters in the late phase with the help of TIC for depicting malignancy and PI for lesion type, thus objectively quantifying washout and wash-in flags. In our study, we evaluated inconclusive FLLs that had been assessed through CEUS, meaning that the analyzed lesions were more difficult to interpret than in a standard situation, even for an experienced eye.

The most prevalent lesion in our analyzed inconclusive FLLs was hepatocellular carcinoma, which accounted for 37% of the lesions; however, if we take into account the regenerative nodules that can be regarded as precursors of HCC, the percentage increases to 45%. HCC was the most challenging lesion in our group and in other relevant studies; consequently, CEUS-based algorithms have been developed in recent years in an attempt to raise the standardization for interpreting and documenting HCC in high-risk patients [23,24,25]. CEUS LI-RADS (contrast-enhanced ultrasound liver imaging reporting and data system) and ESCULAP (Erlanger synopsis for contrast-enhanced ultrasound for liver lesion assessment in patients at risk) are two algorithms that are currently in use for HCC diagnosis. Schellhaas et al. [26] made a direct comparison between the two systems and the regular CEUS interpretation at the time of examination. In cirrhotic patients, ESCULAP had the highest sensitivity in comparison with on-site CEUS and CEUS LI-RADS (94.2% vs. 90.9% vs. 64.9%; *p* < 0.001) and the highest negative predictive value (NPV) (67.4% vs. 62.7%; *p* < 0.001) (67.4% vs. 34.1%; *p* < 0.001); still, the specificity of CEUS LI-RADS was superior to the others. The positive predictive values (PPVs) were comparable between the methods. However, 5.5% of the HCCs were misdiagnosed by both algorithms. The study underlines the value of these algorithms for less-experienced examiners, and with only histology as the gold standard, a bias might appear, since a noninvasive method was evaluated.

In later years, many studies were dedicated to the use of classifiers for designing automated computer assisted diagnosis (CAD), a sort of artificial intelligence (AI) system for medical use [27,28]. In this paper, a binary decision tree classifier (BTDC) was used to assess the considered lesions. This BTDC was demonstrated to be useful for detecting good scores for the benign/malignancy of lesions but with fewer good results when assessing the type of lesion. However, this is a precursor to using machine learning methods and, eventually, neural networks in assessing FLLs.

This study had several limitations. It was a retrospective evaluation of a rather small sample size, where only some of the lesions had histology as a reference, and thus has a possible bias for what concerns the gold standard. Even though the algorithm had good results when comparing it with gold standard methods, it must be validated on a control group in order to avoid an overestimation of the performance in real life. Additionally, the MPUS features used tools imbedded in a single US machine (LOGIQ E9 from GE), which represents an important limitation of the study. Nonetheless, this study also has some strong points. To date, it is the first publication that considers the MPUS strategy for inconclusive FLLs, thus saving time and resources. The MPUS approach might be the recommended approach for liver evaluation. Basic and advanced US features can be fused in an AI-MPUS-based system, which could overcome some of the limitations of US.

## 5. Conclusions

Reassessing inconclusive FLLs that had been evaluated with CEUS using the MPUS algorithm with the help of a tree-based decision classifier, it was possible to establish malignancy in 78% and the lesion type in 28% of the lesions.

## Figures and Tables

**Table 1:** Typical features of the enhancement patterns in CEUS—the arterial phase of the most frequent lesions encountered in daily practice that require CEUS evaluation.

Lesion	Arterial Phase (10–20 to 30–45 s)
Hemangioma (HMG)	Peripheral nodular enhancement
FNH (focal nodular hyperplasia)	Centrifugal hyper-enhancement (possible with feeding artery)
Abscesses (ABS)	Peripheral enhancement possible with septa (no central enhancement—necrotic areas)
Metastasis (Meta)	Rim enhancement or hyper-enhancement
Hepatocellular carcinoma (HCC)	Hyper-enhancement
Cholangiocarcinoma (CC)	Rim-like hyper-enhancement with central hypo-enhancement
Focal fatty change (FFC)	Iso-enhancement

**Table 2:** Patients baseline characteristics.

Parameter	Mean ± SD, *n* (%)
Age	62.3 ± 6.4
Gender (male)	52.1%
Fibrosis stage	
Non-cirrhotic	57 (62.6%)
Cirrhotic	34 (37.4%)
Type of lesion	
Hepatocarcinoma	34 (37.4%)
Metastasis	13 (14.2%)
Haemangioma	7 (7.7%)
Regenerative nodules	7 (7.7%)
Focal liver alterations	5 (5.5%)
Fatty free areas	3 (3.3%)
Cholangiocarcinoma	4 (4.4%)
Abscesses	2 (2.2%
Adenomas	5 (5.5%)
Complex biliary cysts	4 (4.4%)
Parenchymal infarction areas	5 (5.5%)
Vascular abnormalities	2 (2.2%)

SD = standard deviation; *n* = number of subjects.

**Table 3:** Characteristics of the TIC analysis.

Parameter	Lesion	Parenchyma	*p*-Value
A	5.5 ± 21.5	−3.53 ± 61.8	0.98
B	−57.3 ± 21.8	−47.2 ± 59.7	0.72
k	1.5 ± 5.3	0.9 ± 1.3	0.50
TtoPk	7.3 ± 8.0	6.5 ± 7.7	0.89
AREA	−25.0 ± 37.9	−7.0 ± 42.6	0.04

A = the difference between B and the intercept intensity at t = 0; B = the minimum intensity at t = infinity; k = the gradient at t = 0, calculated by “−A*k”; TtoPk = time to peak intensity from the start frame to the end frame; AREA = the area between the start frame and end frame based on start frame intensity.

**Table 4:** Ultrasonography-based performance at differentiating benign vs. malign lesions.

Ultrasonography Techniques	AUROC	*p*-Value	Sensitivity	Specificity	PPV	NPV
B-mode	0.52	0.96	15.6%	84.6%	57.1%	43.4%
Elastography	0.64	<0.001	74.5%	70%	76%	68.3%
CEUS-TIC	0.58	<0.001	74.0%	45.7%	72.5%	64.2%
NEW model *	0.72	<0.001	90.2%	22.5%	59.7%	64.3%

* Composed of CEUS-TIC and elastography.

## Data Availability

The data presented in this study are available on request from the corresponding author. The data are not publicly available due to privacy.
